# Evolving Trends in Female to Male Incidence and Male Mortality of Primary Biliary Cholangitis

**DOI:** 10.1038/srep25906

**Published:** 2016-05-19

**Authors:** Ana Lleo, Peter Jepsen, Emanuela Morenghi, Marco Carbone, Luca Moroni, Pier Maria Battezzati, Mauro Podda, Ian R. Mackay, M. Eric Gershwin, Pietro Invernizzi

**Affiliations:** 1 Liver Unit, Humanitas Clinical and Research Center, Rozzano (MI), Italy; 2 Department of Clinical Epidemiology, Aarhus University Hospital, Aarhus, Denmark; 3 Department of Hepatology and Gastroenterology, Aarhus University Hospital, Aarhus, Denmark; 4 Biostatistic Unit, Humanitas Clinical and Research Center, Rozzano (MI), Italy; 5 Department of Internal Medicine, Policlinico IRCCS San Donato, University of Milan, San Donato Milanese, Italy; 6 Department of Health Sciences, School of Medicine Ospedale San Paolo, Università degli Studi di Milano, Milan, Italy; 7 Department of Biochemistry and Molecular Biology, Monash University, Clayton, VIC 3800, Australia; 8 Division of Rheumatology, Allergy, and Clinical Immunology, University of California at Davis, Davis, CA, USA; 9 Program for Autoimmune Liver Diseases, International Center for Digestive Health, Department of Medicine and Surgery, University of Milan-Bicocca, Milan, Italy

## Abstract

Primary biliary cholangitis (PBC) has been regarded as female-predominant without evidence of gender difference in survival. We aimed to compare the overall survival, incidence and prevalence of PBC in two well defined population-based studies over a recent decade, considering also sex ratios and mortality. We have taken advantage of population-wide records, during 2000–2009, in Lombardia, Northern Italy, and Denmark. We focused on the incident cases of PBC, including gender and outcome, among 9.7 million inhabitants of Lombardia and 5.5 million of Denmark. In Lombardia there were 2,970 PBC cases with a female:male ratio of 2.3:1. The age/sex-adjusted annual incidence of PBC was 16.7 per million. Point prevalence was 160 per million on January 1^st^ 2009. In Denmark there were 722 cases of incident PBC, female:male ratio was 4.2:1, and the annual incidence was 11.4 per million, a point prevalence of 115 per million in 2009. Cox regression multivariate analysis identified male sex as an independent predictor of all-cause mortality in both Italian (HR 2.36) and Danish population (HR 3.04). Our data indicate for PBC a sex ratio significantly lower than previously cited, a reversal of the usual latitudinal difference in prevalence and a surprisingly higher overall mortality for male patients.

Autoimmune diseases include more than 70 different conditions affecting approximately 5% of the population of developed countries^1^; they are a major health problem and can greatly impair the quality of life of affected subjects. Primary biliary cholangitis (PBC), until recently known as Primary Biliary Cirrhosis^2^,^3^,^4^,^5^,^6^,^7^,^8^,^9^, is considered a model autoimmune disease because of the homogeneity among patients and the high disease specificity of the accompanying anti-mitochondrial and antinuclear antibodies (AMA, ANA); there has been an enormous effort in studying PBC in both humans and murine models^10^,^11^,^12^,^13^,^14^,^15^,^16^,^17^,^18^,^19^,^20^,^21^,^22^,^23^. The pathogenesis of PBC remains enigmatic despite the well-established influence of environmental and genetic factors and including extensive immunological data^11^,^23^,^24^,^25^,^26^,^27^,^28^,^29^,^30^. Reliable epidemiological data could suggest clues to etiology by identifying environmental factors, which initiate the disease in predisposed individuals. According to earlier data^24^,^31^,^32^ PBC is considered a disease predominantly affecting women with female:male (F:M) ratios of up to 10:1 and is therefore a prime example of the characteristic sexual dimorphism in autoimmunity. There is a wide variation among studies of incidence and prevalence rates, with rates increasing over time^29^,^32^,^33^. Thus the annual incidence and prevalence rates of PBC per 100,000 inhabitants have ranged from 0.33–5.8 per 100,000 and 1.91–40.2, respectively^34^; attributed to ethnic differences in study populations, methods, and case ascertainment. Most epidemiological studies are performed in developed countries. True population-based studies are scarce, and studies based on case finding are subject to obvious shortcomings which pertain also for other autoimmune diseases^35^. Indeed, there have been multiple attempts to understand the female predominance in a variety of autoimmune and inflammatory diseases^36^,^37^,^38^.

Administrative databases are an alternative data source that should overcome some of the weaknesses evident in previous studies^39^,^40^ and herein we assess data derived from two such databases, for Lombardia, Italy and for Denmark, regions located at latitudinal extremes of continental Europe. Thus we aimed to compare the overall mortality, incidence and prevalence of PBC in two well-defined population-based studies over a recent decade, 2000–2009, considering also sex ratios and mortality. Lombardia, is a Northern Italian province with a population of some 9.7 million, Denmark counts nearly 5.5 million inhabitants.

## Patients and Methods

### Data sources and study population

Two databases were used to identify subjects with PBC and retrieve data, the administrative database of the inpatient population in Lombardia, a Northern Italian province with a population of 9.7 million, and Denmark with a population of about 5.5 million, using the Danish National Patient Registry.

Lombardia accommodates 191 health facilities, including 29 public academic and community hospitals, records over 150 million out-patient visits and provides over 2 million hospital admissions annually. The administrative database contains information on all patients discharged from any hospital in the region, and includes sex, date of birth, discharge diagnoses based on the WHO International Classification of Diseases 9^th^ Edition, Clinical Modification (ICD-9-CM)^41^, dates of hospitalization and discharge, and date and cause of death for patients who died in hospital; data were recorded since 2000. Moreover, all Italian citizens enjoy universal, tax-financed healthcare, enabling access to diagnostic and therapeutic procedures in public hospitals after the charge of a co-payment; subjects affected by chronic diseases, including PBC, can obtain a disease-specific exemption code that frees them from the co-payment. Given that the exemption code frees subjects affected by a chronic disease from the co-payment due for visits and procedures, the number of affected patients that do not request the exemption code is almost nil. Therefore, the exemption code was used in this study to supplement the inpatient registry since it allows identification of PBC patients that never required hospitalization. The study population included subjects over 20 years of age with “Biliary cirrhosis” (571.6) as the primary or secondary diagnosis between January 1^st^, 2000 and December 31^st^ 2009. The date of PBC diagnosis was defined as the date on which patients received their first such diagnosis. Individual information from the register was linked to a serial number by a third party to preserve anonymity. We obtained dates of all cause of death only from in-hospital events since it was not possible to obtain them from the exemption code. Indeed, there is no updating of the exemption data due to death or emigration. Prevalence was calculated matching data from hospital access of PBC patients from 2000 to 2009 and active exemption codes, since the exemption code allowed us to identify PBC patients that were not admitted into a hospital during the study time.

In order to compare our results with those for a different population and jurisdiction, and given that outpatients are not included in the Lombardia administrative database, we accessed the Danish National Patient Registry and identified all Danish citizens diagnosed with PBC during 2000–2009. Denmark has a population of 5.5 million, and the Registry has recorded data from all admissions to Danish non-psychiatric hospitals since 1977^42^. We selected the same information as that included in the Lombardia study, however discharge diagnoses were based on ICD-8 in 1977–1993 and since 1994 on ICD-10. ‘Primary biliary cirrhosis’ was defined by the code K74.3 in ICD-10, so differing from ‘secondary biliary cirrhosis’ (K74.4) and ‘non-specific biliary cirrhosis’ (K74). The date of PBC diagnosis was defined as the date on which patients received their first PBC diagnosis, and patients diagnosed with PBC before 2000 were excluded. Dates of death were obtained from the Danish Central Office of Civil Registration System, which tracks the vital status of Danish residents and is continuously updated^43^.

### Statistical analysis

The incidence rate was defined and computed as the number of patients with a first-time diagnosis of PBC in a particular year divided by the total number of inhabitants at the beginning of that year. For Lombardia, we used a 4 year washout period (2000–2004) to avoid including prevalent cases due to the fact that data recording started in 2000, and determined a 6 year span time (from Jan 1^st^ 2004 to Dec 31^st^ 2009) with no significant variation of the Standardized Incidence Ratio (SIR) (Poisson regression rate P = 0.15). The incidence rate for a range of years was obtained by summing the numerator and denominator for each year. All incidence rates were age-standardized to the WHO Standard Population^41^ with the direct standardization method and 95% confidence intervals (CI) based on the Poisson distribution. The prevalence was computed as the proportion of citizens with PBC divided by the total number of citizens at the beginning of each year, again standardized to the WHO Standard Population. We used the Kaplan-Meier method to estimate survival probabilities, and we used Cox regression to estimate the mortality hazard ratio for men versus women adjusting for age at diagnosis and calendar year of diagnosis, both entered as continuous variables. Relative survival analysis was performed in order to estimate PBC patients’ survival with respect to that of the general population. The analysis was performed using public open access databases reporting general population all causes mortality, including separate data for sex and class of age. All statistical analyses were done using Stata statistical software 12.0 (Stata Corporation, College Station, TX, USA).

## Results

### Incidence, prevalence, and female:male ratio

On Jan 1^st^ 2010 there were 9,742,676 registered residents in Lombardia. Among these, the PBC cohort comprised 2,970 patients of whom 2,073 (69.8%) were female. The mean age at diagnosis was 61.7 (95% CI 61.5–62.7), with a slight, but statistically significant, difference between males (60.8 years) and females (62.1 years). A 2.3:1 F:M ratio was observed for this PBC cohort. In Denmark, among 5,534,738 residents, there were 722 PBC cases identified, 584 females and 138 males in the 10-year observation time with the F:M ratio being 4.2:1. The mean age at diagnosis was 60.7 years, 59.3 in males and 61.1 in females.

**Figure 1:**
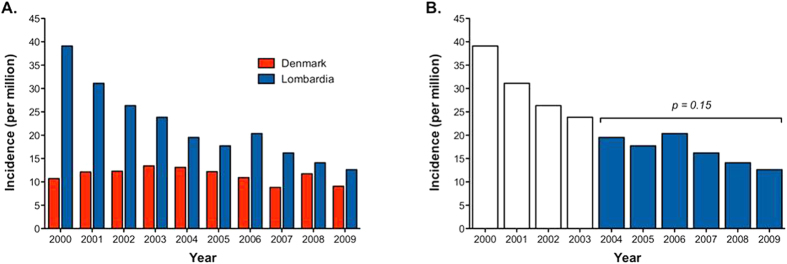
Standard incidence rate in Lombardia and Denmark. (**A**) the trend between both populations was not homogeneous as confirmed by the regression model applied (Lombardia Poisson regression rate P < 0.0005, Denmark Poisson regression rate P = 0.48). (**B**) We therefore assume a 4-year washout in order to avoid SIR overestimation and determined a six year span time (from Jan 1^st^ 2004 to Dec 31^st^ 2009) with no significant variation of SIR (P = 0.15).

SIRs of PBC for Lombardia, standardized on a WHO Standard Population^41^, was 16.7 per million/year during 2004–2009, compared to 11.4 million/year in Denmark (Table 1A). However the trend between both populations was not homogeneous as confirmed by the regression model applied (Lombardia Poisson regression rate p < 0.0005, Denmark Poisson regression rate p = 0.48) (Fig. 1A). We hypothesize that, since no administrative data in Lombardia were available before 2000, in the first years after 2000 there would be a mix of truly incident patients and patients who were in fact diagnosed with PBC before 2000. We therefore assume a 4-year washout was needed in order to avoid SIR overestimation and determined a six-year span time (from Jan 1^st^ 2004 to Dec 31^st^ 2009) with no significant variation of SIR (p = 0.15) (Fig. 1B). The sex-specific SIR shows that the F:M ratio remained constant over the studied decade in both Lombardia and Denmark (data not shown).

**Figure 2:**
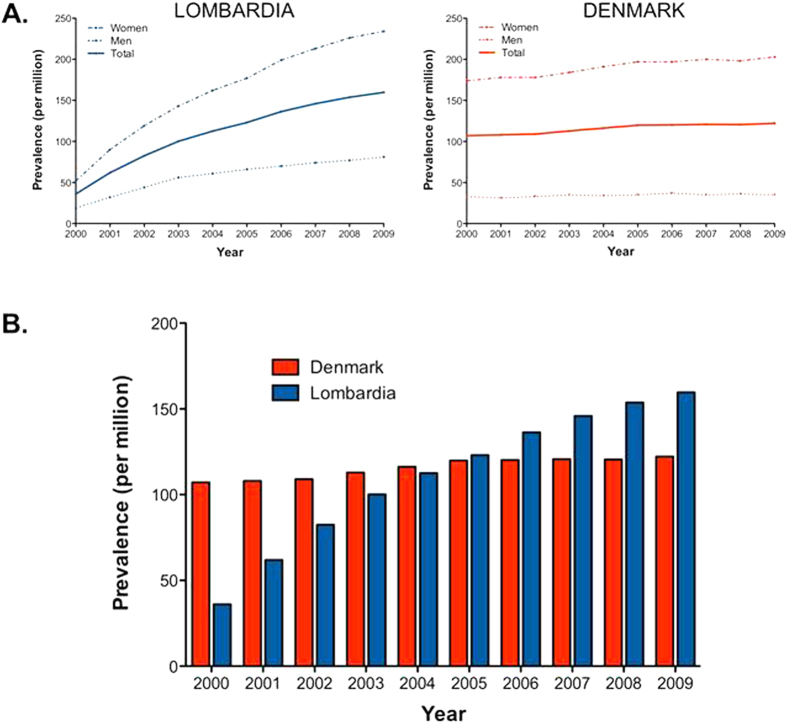
PBC point prevalence in Lombardia and Denmark from 2000 to 2009. The figure shows that whereas point prevalence remains constant during the study time in Denmark (Poisson P = 0.12), it increases from 2000 to 2009 (P < 0.0005) **(A,B)**. This dynamics is observed in both male and females **(A**).

The mean point prevalence of PBC in Lombardia in 2009 was 160 cases per million (234 female, 81 male) Table 1B. The prevalence in 2009 seems to be the most reliable one for Lombardia since the rate did not reach a plateau during the follow-up period (Fig. 2). Indeed, whereas point prevalence remains constant during the study time in Denmark (Poisson P = 0.12), it increases from 2000 to 2009 (P < 0.0005) (Fig. 2A,B). Such dynamics seem to be related to the method in use for diagnosis identification. In Lombardia a diagnosis of PBC was made at admission to the hospital starting from 2000 whereas Danish registries recorded a prevalent diagnosis even if it was made before 2000. In order to avoid this limitation we put together the exemption code registry in Lombardia and the inpatient registry data, eliminate duplicates, and calculated the point prevalence in 2010 which was 295 cases per million (486 female, 159 male)

### Survival and prognostic factors: males fare badly/do worse

**Figure 3:**
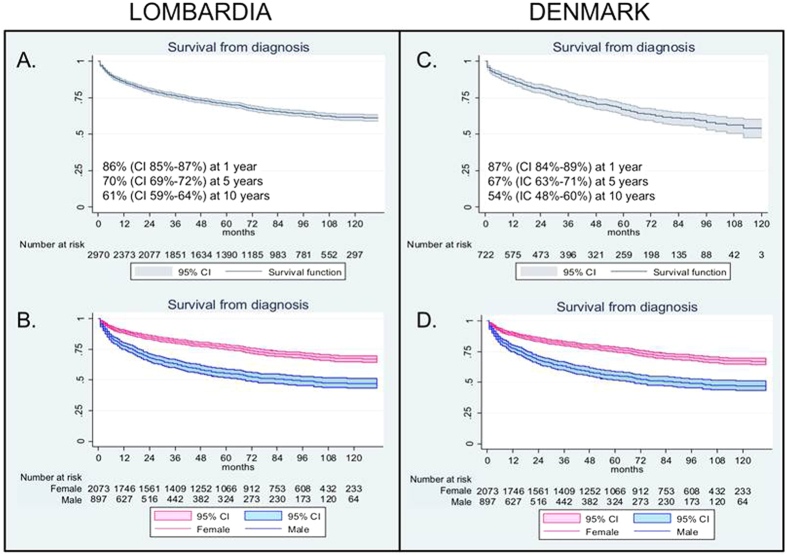
Survival and sex specific survival from diagnosis in Lombardia (**A,B**) and Denmark (**C,D**). The figure shows higher mortality of PBC male patients in both populations. CI of 95%.

**Figure 4:**
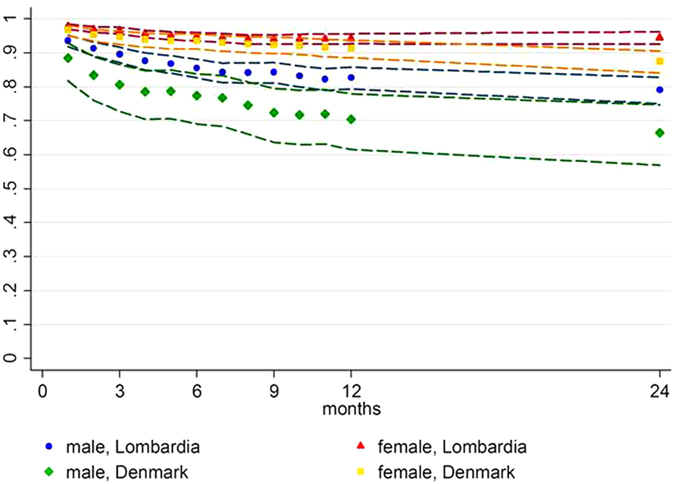
Relative survival rates (RSR) for PBC, by sex in both Lombardian and Danish population. The figure shows lower RSR of PBC male patients in both populations, all the groups show RSR < 1. Bands indicate 95% CI.

2,970 incident PBC cases in Lombardia were observed for a mean follow-up of 4.8 years, and 14,446 person-year from PBC diagnosis. The 1, 5 and 10 year survival rate estimation was respectively 86% (CI 85–87%), 70% (CI 69–72%) and 61% (CI 59–64%) (Fig. 3A). The sex specific survival at 1, 5 and 10 years was significantly higher for females (n = 2,073), respectively 89% (CI 88–91%), 77% (CI 75–78%) and 67% (CI 65–70%) than for males (n = 897), respectively 78% (CI 75–80%), 55% (CI 52–59%) and 47% (CI 43–51%) (Fig. 3B). In Denmark a survival analysis was done by following 722 incident cases for 2,780 person-years from PBC diagnosis, with an mean follow up of 3.9 years. Survival rate estimates, at 1, 5 and 10 years, were similar to those observed in Lombardia, respectively 87% (CI 84–89%), 67% (CI 63–71%) and 54% (CI 48–60%) (Fig. 3C). The sex specific survival rate in Denmark resembled the higher mortality in men with PBC observed in Lombardia since, in Denmark, survival of females at 1, 5 and 10 years was 90% (IC 87–92%), 73% (CI 69–77%) and 60% (CI 53–67%) and in males, 72% (CI 63–79%), 42% (CI 32–51%) and 27% (CI 14–43%) (Fig. 3D). Most importantly the relative survival analysis demonstrated that the higher mortality in men with PBC was independent of the higher male mortality in the general population (Fig. 4).

Using the Cox proportional hazard model, including sex, age at diagnosis and year of diagnosis (Table 2), male sex was associated with a higher risk of all-causes death for both populations: Hazard Ratio (HR) 2.36 in Lombardia, and 3.04 in Denmark.

## Discussion

In the present study we report the epidemiology and natural history of PBC for two populations, Lombardia and Denmark, reflecting two diverse latitudes of continental Europe. Our findings include a significantly lower than hitherto cited F:M ratio for both the Italian and Danish populations, a probable decrease in incidence over time (particularly for Lombardia), a lack of any latitudinal prevalence gradient, and a surprisingly higher overall mortality for male than female PBC patients for both populations.

One strength of our study is the use of population-based databases, which obviates the selection bias inherent in studies restricted to just a single or a few health facilities or reference centers. The use of administrative data has been well validated for the Danish model, ensuring registration of any event within the health system of defined geographical boundaries^40^.

Previous epidemiological research on PBC, mainly European, has reported incidence rates ranging from 2 to 49 cases per million/year and prevalence of between 19 and 402 cases per million (Table 3)^32^; our data fit within the wide range reported and are significantly different among them. Of note, our data showed that the trend between both populations was not homogeneous with an apparent decrease in incidence over time especially in Lombardia. We hypothesize that, since no administrative data in Lombardia were available before 2000, in the first years after 2000 there would be a mix of truly incident patients and patients who were in fact diagnosed with PBC before 2000. Indeed, assuming a 4-year washout was needed in order to avoid SIR overestimation, no significant variation of SIR was determined.

On the other side, the much larger number of diagnoses of PBC in Lombardia may be explicable by a heightened awareness of physicians to liver diseases since, in Italy as in other Mediterranean countries, there is a higher prevalence of viral hepatitis. Moreover, the heterogeneity of epidemiological data can be the result of demographic changes, increasing survival, earlier diagnosis, improved treatment and greater recognition of the disease by physicians and patients^32^. Furthermore, the diagnostic rate of PBC may have overall increased during these years.

The ratio of females to males with PBC in Lombardia was 2.3:1, lower than that observed in Denmark (4.2:1); however, in both cohorts the female:male ratio was far lower than any other sex ratio previously reported (Table 3). Of note, two other large epidemiological studies based on administrative data demonstrate a higher than expected presence of male PBC patients (Table 4)^44^,^45^. One of these is a recent paper comprising data from 33 different autoimmune disease patients from administrative databases in Sweden^44^. The authors included 2,852 PBC patients, 1,080 of which were male, showing a female: male ratio of 1.6:1, in accordance with the trend revealed by our data and other population based studies^45^. Data obtained from reference centers demonstrate that prevalence is strongly biased to females (9:1), possibly influenced by the greater use by women of medical check-ups in highly specialized centers. The present analysis of population-wide registers, however, reflects a muted difference in the frequency of PBC between the sexes more in conformity with that seen in other autoimmune diseases.

Importantly, besides the differences in incidence and prevalence in Northern Italy and Denmark, the relatively higher mortality in male PBC patients was consistent in both datasets. Indeed, male sex was confirmed to be an independent predictor of all-cause mortality in both populations. This finding has been reported once^46^, but has not been generally replicated^47^,^48^; however, the Canadian epidemiological study^45^ did report male sex as an independent predictor of mortality (HR 5.06). Several hypotheses can be offered for the causes of increased male mortality, including lack of compliance, additional environmental exposures and unknown sex factors that may modulate immunity. Thus, Carbone *et al*. in a recent study from UK, showed that men were significantly less likely to have responded to ursodeoxycholic acid (UDCA) than women; male sex was an independent predictor of nonresponse on multivariate analysis^49^. Moreover, Carbone and colleagues reported than men were less likely to be symptomatic, which may be a cause of delay in diagnosis^49^. Unfortunately no data regarding symptoms, use of UDCA and adherence to therapy were available in our database; similarly no clinical data were available in our database that would have helped to ascertain whether liver-related deaths were more frequent. We used the need for liver transplant as a surrogate endpoint for liver related death, but only 39 PBC patients in Demark and 58 in Lombardia were transplanted during the decade of study, suggesting that there was no association.

Our study has some weaknesses that could, at least in part, explain the reported differences between the two included populations. First, the diagnosis code used in Lombardia (ICD-9-CM 571.6) identifies cases of biliary cirrhosis including therefore cases of secondary biliary cirrhosis. Unfortunately, with ICD-9 secondary biliary disease that cannot be excluded, which reduces the specificity and may explain the higher number of cases of PBC reported in Lombardia. Of note, primary sclerosing cholangitis (PSC) has its own code in the Italian system and, therefore, no PSC patients were included. Second, a non-coded diagnosis of PBC at release from the hospital, as well as among outpatients with no exemption code would not be identified in the Italian database whereas the Denmark Registry does include that information. Third we obtained dates of death only from in-hospital events since it was not possible to obtain these from the exemption code. This will probably lead to underestimation of overall mortality, however it will not change the male: female ratio.

In conclusion, our population studies based on disease registers questions some long-held beliefs on determinants of PBC. Females, although more frequently affected by PBC, seem to have a better outcome. Our findings clearly define an important clinical message for the hepatologist: male PBC patients have higher mortality than their female counterparts, such that close clinical follow-up and checking adherence to therapy are strongly recommended. In addition, a steadily increasing prevalence in Lombardia points to possible unsuspected environmental effects in causation, at least in that population, although biased estimation due to factors like increasing awareness of the disease or increasing hospitalization of patients cannot be excluded. Finally, the cause(s) of increased male mortality in PBC calls for a larger and prospective study and should be addressed in the future with well-designed observational trials.

## Additional Information

**How to cite this article**: Lleo, A. *et al*. Evolving Trends in Female to Male Incidence and Male Mortality of Primary Biliary Cholangitis. *Sci. Rep.* **6**, 25906; doi: 10.1038/srep25906 (2016).

## Figures and Tables

**Table 1:** (A) Standardized incidence rate in Lombardia and Denmark.

A							
LOMBARDIA				DENMARK			
Year	Female	Male	Total (95% CI)	Year	Female	Male	Total (95% CI)
2000	55.4	21.9	39.1 (35.5–42.7)	2000	17.6	3.5	10.7 (8.0–13.4)
2001	41.0	20.4	31.1 (27.9–34.3)	2001	18.2	5.5	12.1 (9.3–14.9)
2002	34.1	17.2	26.4 (23.4–29.3)	2002	19.5	4.6	12.3 (9.5–15.1)
2003	30.5	16.3	23.9 (21.0–26.7)	2003	21.9	4.4	13.4 (10.5–10.5)
2004	25.7	13.1	19.5 (17.0–22.0)	2004	20.0	6.2	13.1 (10.3–16.0)
2005	21.7	12.8	17.5 (15.2–20.2)	2005	18.2	5.9	12.2 (9.5–14.9)
2006	27.9	11.4	20.4 (17.7–23.1)	2006	16.3	5.1	10.9 (8.3–13.6)
2007	21.1	9.8	16.2 (13.8–18.6)	2007	14.2	3.3	8.4 (6.5–11.2)
2008	18.7	9.1	14.1 (12.0–16.2)	2008	18.7	4.3	11.7 (9.1–14.4)
2009	16.1	8.3	12.6 (10.5–14.7)	2009	13.3	4.9	9.1 (6.8–11.4)
2004–09	21.9	10.7	16.7 (14.4–19.1)	2000–09	17.7	4.7	11.4 (10.6–12.3)
**B**							
2000	52	19	36 (33–40)		174	33	107 (99–115)
2001	90	32	62 (57–66)		178	31	108 (100–116)
2002	119	44	82 (77–88)		178	33	109 (101–117)
2003	143	56	100 (94–106)		184	35	113 (104–121)
2004	162	61	113 (106–119)		191	34	116 (108–125)
2005	177	66	123 (117–129)		197	35	120 (111–128)
2006	199	70	136 (129–143)		197	37	120 (112–129)
2007	213	74	146 (139–153)		200	35	121 (112–129)
2008	226	77	154 (146–161)		198	36	120 (112–129)
2009	234	81	160 (152–167)		203	35	122 (114–131)
2000–2009	162	58	111 (109–113)		190	34	115 (113–118)

For Lombardia, we used a 4 year washout period (2000–2004) to avoid including prevalent cases and determined a 6 year span time (from Jan 1^st^ 2004 to Dec 31^st^ 2009) with no significant variation of the Standardized Incidence Ratio (SIR). (**B**) Point prevalence of PBC in Lombardia and Denmark from 2000 to 2009.

**Table 2:** Cox regression model for predictors of mortality in PBC (2000–2009).

Factor	LOMBARDIA	DENMARK
	Hazard Ratio (95% CI)	Hazard Ratio (95% CI)
Male sex	2.36 (2.08–2.69)	3.04 (2.31–4.01)
Age at diagnosis	1.06 (1.06–1.07)	1.07 (1.06–1.08)
Year of diagnosis	1.29 (1.24–1.34)	1.19 (1.11–1.29)

**Table 3:** Epidemiological studies on PBC.

Area	Year	Patients (n)	Prevalence (per 10^6^)	Incidence (10^6^/year)	Age (years)	Sex rate (F:M)
Europe^50^	1984	569	23	54	54	10:1
Sweden^51^	1985	111	151	13,3	55	6:1
Newcastle, UK^52^	1989	347	154	19	58	9:1
Ontario, Canada^53^	1990	225	22	3,3	59	13:1
Estonia^54^	1995	69	27	2,3	–	22
Newcastle, UK^55^	1997	160	240	22	66	10:1
Norway^56^	1998	21	146	16	–	9:1
Minnesota, USA^57^	2000	46	402	27	–	8:1
Newcastle, UK^58^	2001	770	251	31	–	10:1
Victoria, Australia^59^	2004	249	51	–	61	9:1
Japan^60^	2005	9761	78	–	–	9:1
Canada^45^	2009	137	227	30	53	5:1
Denmark	2011	722	115	11.2	61	4.2:1
Lombardia	2011	2970	160	16.7	62	2.3:1

**Table 4:** Population based epidemiological studies demonstrating high female:male ratio in PBC.

Area	Years (range)	Subjects included	Sex rate (F:M)	Ref.
Canada	1996–2002	1,100,000	4.8:1	45
Sweden	1964–2008	535,538	1.6:1	44
Denmark	2000–2009	5,500,000	4.2:1	–
Lombardy (Italy)	2000–2009	9,700,000	2.3:1	–

Two large epidemiological data sets are compared to the data obtained from administrative data in Lombardy (Italy) and Denmark and reported in the present study.
